# Transient ocular surface non-perfusion during cataract phacoemulsification surgery

**DOI:** 10.1186/s12886-020-01538-2

**Published:** 2020-07-31

**Authors:** Yijun Hu, Wei Qi, Baoyi Liu, Honghua Yu

**Affiliations:** 1 Aier Institute of Refractive Surgery, Refractive Surgery Center, Guangzhou Aier Eye Hospital, Guangzhou, China; 2 Aier School of Ophthalmology, Central South University, Changsha, China; 3 Department of Ophthalmology, Guangdong Provincial People’s Hospital, Guangdong Academy of Medical Sciences/The Second School of Clinical Medicine, Guangdong Eye Institute, Southern Medical University, Guangzhou, 510080 China

**Keywords:** Cataract surgery, Phacoemulsification, Complication, Case report

## Abstract

**Background:**

Complete non-perfusion of the anterior segment vasculature during cataract phacoemulsification surgery is rarely reported.

**Case presentation:**

We present two cases of transient ocular surface non-perfusion (TOSN) during cataract phacoemulsification surgery. The TOSN happened during intraocular lens (IOL) implantation with complete vanish of blood flow in the conjunctival, episcleral and limbal vessels. Reperfusion started within 30 s and part of the blood supply of the conjunctiva and episclera was restored within 2 min. However, the blood flow in the limbal vessels was not restored until 5 min later. The postoperative examination of both eyes was normal. These two rare cases are the TOSN during cataract surgery. We speculated that the significantly increased intraocular pressure during IOL implantation might be the cause of this rare phenomenon.

**Conclusion:**

Non-perfusion of the ocular structures may occur if the IOP peak during IOL injection exceeds the perfusion pressure of the anterior vasculature.

## Background

Vascular complications of the anterior segment during cataract phacoemulsification surgery are not common. Some complications are due to accidental damage of the anterior segment tissue by surgical instrumentation or careless maneuver. Complications such as subconjunctival hemorrhage and hyphema have been reported [1, 2]. However, report about complete non-perfusion of the anterior segment vasculature in the literature is rare. In this report, we present two cases of transient ocular segment non-perfusion (TOSN) during cataract phacoemulsification surgery.

## Case presentation

**Fig. 1:**
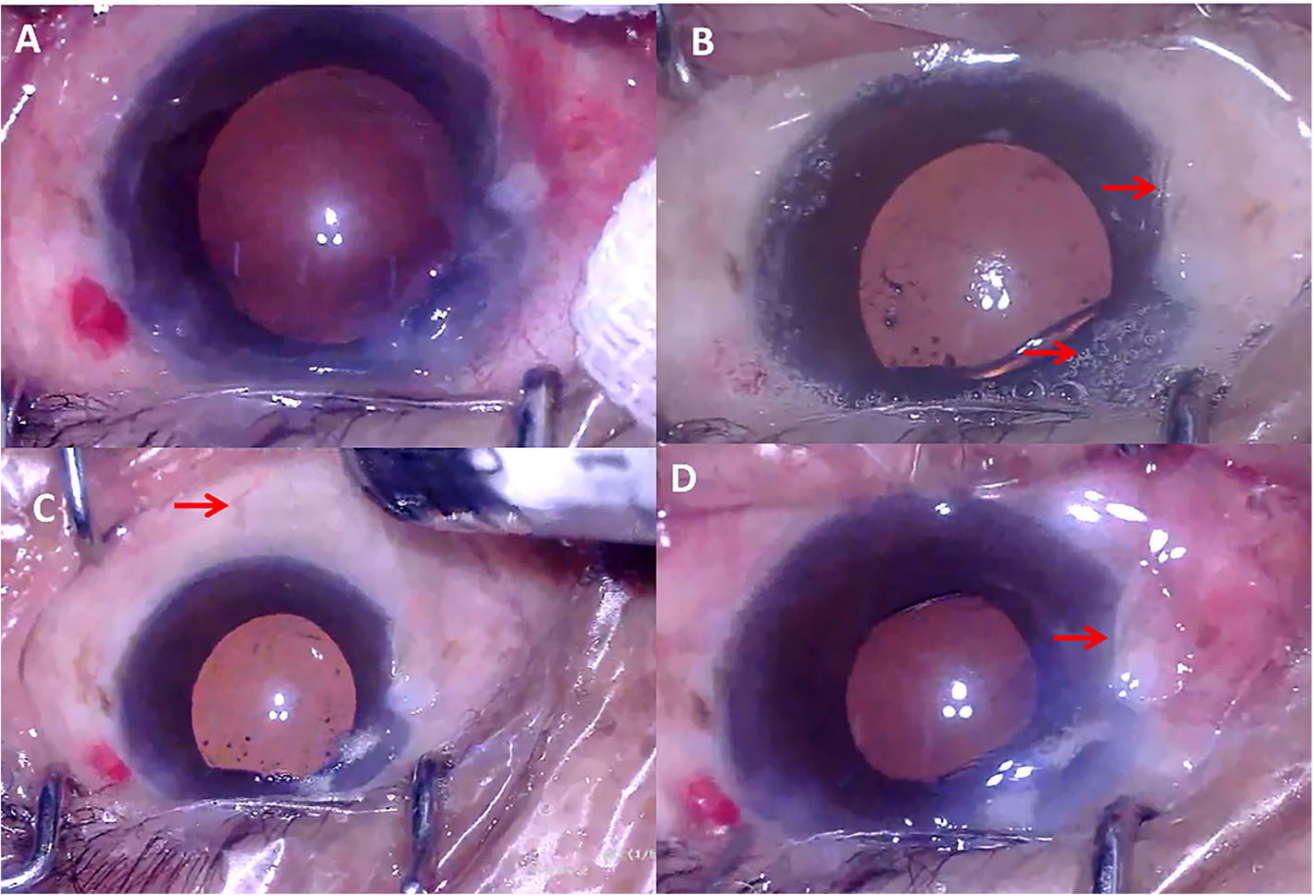
Demonstration of the TOSN in patient 1. **a** Before non-perfusion. **b** On the time of non-perfusion. Notice the gas bubbles and pale pterygium (arrow). **c** The reperfusion started. Notice the episcleral artery (arrow). **d** Reperfusion of the majority of conjunctival and episcleral vessels. Notice the color to the pterygium (arrow)

**Fig. 2:**
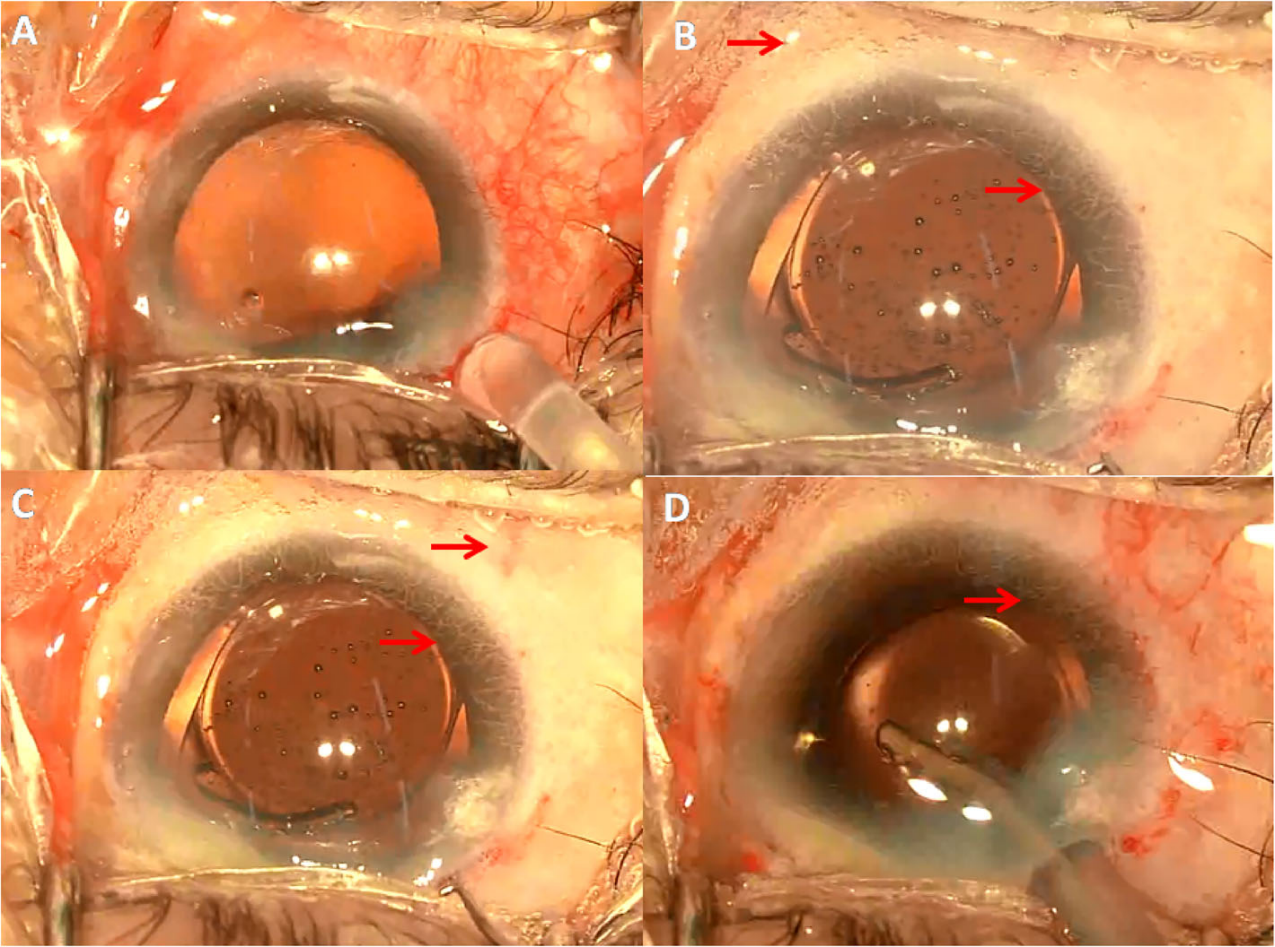
Demonstration of the TOSN in patient 2. **a** Before non-perfusion. **b** On the time of non-perfusion. Notice the gas bubbles and limbal vessels (arrow). **c** The reperfusion started. Notice the episcleral artery and limbal vessels (arrow). **d** Reperfusion of part of the conjunctival and episcleral vessels. Non-perfusion of the limbal vessels still present (arrow)

The first case was a 77 years old male (patient 1) and the second case (patient 2) was a 66 years old female. They both underwent scheduled phacoemulsification surgery for senile cataract (left eye in patient 1 and right eye in patient 2). The ocular and systemic examination and history of the two patients were unremarkable (except for cataract, patient 1 also had nasal pterygium). Cataract phacoemulsification surgery was scheduled and performed under topical anesthesia using 0.5% proparacaine hydrochloride eyedrops (Alcaine, Alcon) (patient 1 by Y.H. and patient 2 by W.Q.). A superonasal (patient 1) or superotemporal (patient 2) clear corneal incision (CCI) was made. Manual continuous curvilinear capsulorhexis followed by hydrodissection and hydrodelineation was proceeded. The nucleus was removed by phacoemulsification and residual cortex was removed by irrigation/aspiration with the infusion bottle height of 90 cm in both procedures. The capsular bag and anterior chamber were filled with cohesive ophthalmic viscoelastic device (OVD) (1.7% Medical Sodium Hyaluronate Gel, Bausch & Lomb-Freda). The amount of OVD was determined by satisfactory expansion of the capsular bag while maintaining a moderate intraocular pressure (IOP). A single-piece hydrophilic acrylic foldable intraocular lens (IOL) was implanted through the CCI by an injector and that was when the conjunctiva non-perfusion happened. Right on the time of IOL injection (in patient 1) or right after the IOL was injected (in patient 2), the blood flow in the bulbar conjunctival, episcleral and limbal vessels diminished rapidly with appearance of small gas bubbles on the surface of the bulbar conjunctiva. The gas bubbles emerged from all of the bulbar conjunctiva. The color of the ocular surface turned pale in the following 2–5 s. The patients’ vitals were checked and were normal and their blood pressure during the surgeries was within normal range. The blood flow spontaneously started to perfuse the episcleral arteries 20 s later, and complete perfusion in part of the conjunctival and episcleral vessels were seen 2 min later (Fig. 1 and Fig. 2). However, the blood flow in the limbal vessels was not restored until 5 min later. Postoperative examination [visual acuity, IOP, corneal condition, anterior chamber reaction] of both eyes did not reveal significant difference compared to the other eyes underwent cataract surgery on the same day.

## Discussion and conclusion

These two rare cases are the TOSN during cataract surgery. For the reason of the TOSN, we have ruled out the possibility of vascular constriction caused by OVD and IOL preservative fluid, because under manufactured concentration none of their components could have caused severe vessel constriction as in our cases. To better understand the process of the TOSN we reviewed the vascular supply of the bulbar conjunctiva, episclera and limbus. The anterior ciliary arteries (ACAs) provide the majority of blood supply to the episclera, limbus and anterior bulbar conjunctiva. The ACAs originate from the rectus muscles [3]. After exiting the muscles, they run within the Tenon’s capsule and later give rise to the episcleral and scleral branches. The episcleral branches of the ACAs run anteriorly to form the limbus vasculature and supply the bulbar conjunctiva within 4 mm from the limbus. The bulbar conjunctiva also receives a second blood supply from the palpebral arteries [4]. Running closely to the sclera in their paths, the ACAs are easily affected by changes in the sclera.

In our cases, the TOSN happened during IOL implantation and we speculated that it was a process with multiple stages. Based on the deformation of the eyes during the insertion of the IOL injector and adjustment of the position of the IOL, the IOP was not significantly increased before or after IOL injection. However, the IOP was very likely to be significantly elevated during IOL injection, based on the sudden hardening of the eyeballs and the increased resistance the surgeons felt. The elevated IOP during IOL injection might be the initial cause of conjunctiva non-perfusion. In fact, the change of IOP during IOL implantation through a 1.9–2.5 mm incision has been documented in previous studies on cadaver or live eyes [5–7]. Kamae et al. recorded a biphasic pattern of IOP elevation during IOL injection. The first phase of IOP elevation occurred during the optic entering the eye and the second phase occurred when the trailing haptic was pushed into the eye [5]. Kamae et al. also found that the peak IOP during IOL injection might as high as 115 mmHg (double of the retinal perfusion pressure) in a successful attempt and 306 mmHg in an unsuccessful attempt [5]. We supposed that the TOSN might occur when the peak IOP during IOL injection exceeded the perfusion pressure of the conjunctival and episcleral arterioles. The significant IOP elevation during IOL injection could cause a significant increase in rigidity and bulging of the sclera. The significantly harden and bulged sclera in turn compressed the ACAs and their episcleral branches that provided arterial supply to the conjunctiva, episclera and limbus. The non-perfusion also caused subsequent closure of the capillary bed of the ocular surface vessels. We are not sure about the mechanisms of how the gas bubbles appeared on the surface of the bulbar conjunctiva during TOSN. However, a previous study has shown that in ischemic stroke, multifocal blood-brain barrier disruption may occur around capillaries/venules [8]. We speculate that the gas bubbles might be the oxygen/carbon dioxide released from the capillaries/venules of the bulbar conjunctiva whose blood barrier was disrupted during TOSN. With the IOP dropping from the peak after IOL injection, the blood supply from the ACAs and the episcleral branches was restored gradually. With reperfusion of the capillary bed, the color of the ocular surface turned pinker accordingly. Since the bulbar conjunctiva received a second blood supply from the palpebral arteries, reperfusion of bulbar conjunctiva was faster than the limbus. Moreover, the capillaries of the limbus are embedded in the corneal collagen and have thicker endothelium than the bulbar conjunctiva [9]. Edema of the corneal stroma and capillary endothelial cells might also contribute to the delayed reperfusion of the limbus [10].

In conclusion, although the exact mechanisms of the TOSN warrant further investigation, these two cases should raise our attention to the IOP increase during IOL injection. Non-perfusion of the ocular structures may occur if the IOP peak during IOL injection exceeds the perfusion pressure of these ocular structures. Although ocular ischemic vascular events during routine cataract surgery are rare, particular attention should be paid to the patients with compromised ocular vascular supply. Less OVD filling, lower bottle height, and implantation of the IOL through a larger incision may be considered in these patients during routine cataract surgery.

## Supplementary information

**Additional file 1.**

## Data Availability

The data used during the current study are available from the corresponding author on reasonable request.

## Abbreviations

TOSN: Transient ocular surface non-perfusion
IOL: Intraocular lens
CCI: Clear corneal incision
OVD: Ophthalmic viscoelastic device
ACAs: Anterior ciliary arteries
IOP: Intraocular pressure
